# Supplementary material for: Symptoms, Treatment, and Outcomes of COVID-19 Patients Coinfected with Clostridioides difficile: Single-Center Study from NE Romania during the COVID-19 Pandemic
Source: Antibiotics (Basel). 2023 Jun 22;12(7):1091. doi: 10.3390/antibiotics12071091 (PMC10375993; doi:10.3390/antibiotics12071091)
Supplement: Supplementary file 1 [file antibiotics-12-01091-s001.zip › antibiotics-2439351-supplementary.pdf]

antibiotics

MDPI

Supplementary Materials

Table S1. Associations between comorbidities and COVID-19 severity in the coinfected patients group (*N* = 86).

Comorbidities	COVID-19 Severe form of disease (*N* = 36)	COVID-19 Mild/moderate form of disease (*N* = 50)	*t*- value	*p*-value
Cardiovascular	52.7%	58%	−0,476	0.635
Diabetes	61.1%	10%	53.69	<0.01
Pulmonary	5.5%	2%	0.816	0.381
Obesity	22%	16%	0.725	0.470
Neurologic	13.8%	20%	−0.730	0.467
Psychiatric	11.1%	2%	1.6	0.115
Endocrine	0%	6%	49	0.83
Chronic Kidney Disease	8.3%	10%	84	0.796
Oncologic	13.8%	12%	84	0.138
Dialysis	0%	6%	*49*	0.083

Table S2. Associations between symptomatology and patients gender in the coinfected patients group (*N* = 86).

Symptomatology	Male *N* = 45	Female *N* = 41	*t*-value	*p*-value
Fever	51%	54%	0.234	0.816
Shivers	40%	24%	1.546	0.126
Productive cough	33%	32%	0.030	0.976
Dry cough	22%	22%	2.049	0.044
Asthenia	53%	32%	2.055	0.043
Myalgia/arthralgia	56%	59%	83.31	0.976
Odynophagia	18%	27%	74.51	0.390
Headache	7%	12%	84	0.142
Anosmia	2%	34%	79.67	0.624
Ageusia	9%	12%	74.51	0.390
